# Children and adolescents in institutional care versus traditional families: a quality of life comparison in Japan

**DOI:** 10.1186/s12955-018-0980-1

**Published:** 2018-07-28

**Authors:** Takahiro Nakatomi, Shuhei Ichikawa, Hideki Wakabayashi, Yousuke C. Takemura

**Affiliations:** 1 Department of Family Medicine, Mie University Graduate School of Medicine, 2-174 Edobashi, Tsu, Mie 514-8507 Japan; 2 Department of Family Medicine, Graduate School of Medical and Dental Sciences, Tokyo Medical and Dental University, 1-5-45 Yushima, Bunkyo, Tokyo, 113-8510 Japan

**Keywords:** Quality of life, Emotional well-being, Child neglect, Family, Orphanages, Residential facilities

## Abstract

**Background:**

A good quality of life (QOL) is important for the physical and mental well-being of all children. However, young people who live in an institutional setting may face different challenges than those who are raised in a traditional family. While a few quantitative studies of institutionalized children’s QOL have been conducted, no research has investigated the QOL of young people living in Children’s Homes (CHs) in Japan. This research compared the QOL of children and adolescents in Japan who live in CHs with that of children and adolescents living in traditional families.

**Methods:**

A cross-sectional study was conducted in July 2016 with 204 students (grades three through nine), 47 of whom lived in a CH, and 157 of whom lived in a traditional family. Ages ranged from 8 to 15 years (CH: 55.8% Female, 44.2% Male; Traditional: 54.1% Female, 45.9% Male). Participants answered the kid-Kinder Lebensqualität Fragebogen (Translated from German: Children’s quality of life questionnaire; KINDL®) Japanese Version, which covers six subscales of QOL; they filled in the questionnaires at home. Analysis of variance was used to compare QOL between the two samples.

**Results:**

The total QOL score for all students (combined elementary school students and junior high school students) from CHs was statistically significantly lower than that for students from traditional families. Scores for the subscales, emotional well-being and family, were also significantly lower for CH young people than for those in traditional families. While elementary pupils in CHs reported lower QOL than those in traditional families, no significant differences in QOL were seen between junior high school students from CHs and their peers from traditional families.

**Conclusions:**

The findings presented support previous research showing that the QOL of elementary school students living in CHs is significantly lower than that of their peers in traditional families. However, this difference was not observed among junior high school students. This contrast suggests that QOL changes with age. Future research is needed to evaluate the determinants of QOL among all generations and family contexts.

## Background

Child abuse is a serious social problem worldwide, including in Japan. The number of recognized child abuse cases in Japan was 103,260 in 2015, which is the highest on record in that country [1]. Approximately 26,500 children between the ages of 3 and 18 currently live in 615 Children’s Homes (CHs) in Japan [2]. CHs were established based on Japan’s child social welfare laws in 1947. They house orp haned, neglected, or abused children; 60% are abused [3]. Institutional care is categorized into large CHs (58%) and small CHs (42%) [4, 5]. The capacity of large CHs is 35 to 50 children, while that of small CHs is six children. The number of foster children in Japan is only 4731, which is approximately 12% of children in need of social care [2]. The Child Guidance Center is a special child welfare organization established under Article 12 of the Child Welfare Act. According to Article 33 of the Child Welfare Law, the Child Guidance Center has the legal authority to separate parent and child. Children raised in CHs are protected by the Child Guidance Center.

The United Nations (UN) has recommended that Japanese governments provide social care for children in need in family-like settings, such as foster families or small group settings in residential care [6]. The UN recognizes that large institutional care can complement family-based care and meet the needs of children to some extent.

However, the UN has also declared that alternatives should be developed in the context of an overall deinstitutionalization strategy, with precise goals and objectives. They encourage states to take the guidelines of the alternative care for children in family-like environments into account, and to bring those guidelines to the attention of the relevant executive, legislative, and judiciary bodies of government [7]. As a previous study of Romanian orphans showed, institutional care is not appropriate for children, as they often have, for example, behavioral and developmental problems [8]. Indeed, abused children living in CHs in Japan show more behavioral and emotional problems than non-abused children in CHs [9]. Furthermore, many abused children live together in CHs and thus experience behavioral and interactional challenges such as fights and arguments [10].

Today, institutional care is less common in Europe and North America [11]; children in out-of-home care are mainly in foster care. Indeed, many countries are moving from institutional care to foster care [12]. However, no previous study has compared children living in CHs with those living in traditional families in Japan. The authors’ understanding of a traditional family in this study is that of children and parents living together in their own home.

In this study, Quality of Life (QOL) is a measure of comprehensive life satisfaction that combines an individual’s feelings of satisfaction about: their physical and mental health, human relationships, family, education, safety, freedom, and the environment [13]. QOL serves as an indicator in policy evaluation, and improving children’s QOL is regarded as important from the viewpoint of children’s human rights [14]. Brady & Caraway [15] point out, however, that “while recent research has focused on the impact of abuse and other interpersonal traumas in childhood, little attention has been given to the experiences of children who have been removed from their homes.” Furthermore, until recently, QOL has not received much attention in the child abuse literature, and the QOL of children institutionalized in CHs in Japan has not been studied [16]. Evaluating the QOL of children is an important societal issue to help develop policies for and determine the allocation of social resources [16].

The purpose of this study was to compare the QOL of children living in CHs with those of children living in traditional families in Japan. Additionally, we compared the QOL of children living in large versus small CHs in Japan.

## Methods

### Participants

The total number of participants recruited for this study was 216; data from 204 of these students, who were in third through ninth grade, were analyzed. Of these, 47 lived in a CH, and 157 lived in a traditional family. We gave 56 questionnaire booklets to CHs and collected 48 completed questionnaires. Additionally, we distributed 243 questionnaire booklets to schools and collected 168 completed questionnaires. Thus, approximately 86% of CH children and 69% of traditional family children and their parents agreed to participate in the questionnaire survey. One child from a CH and 11 children from traditional families made mistakes in their responses, so those questionnaires were eliminated. Ages ranged from 8 to15 years (CH: 55.8% Female, 44.2% Male; Traditional: 54.1% Female, 45.9% Male). First and second grade students were excluded from the study because they are more likely to need help from adults when answering a questionnaire. CH residents were recruited from six CHs, including two large and four small CHs, in Mie and Aichi prefectures in Japan (CH group). The large CHs housed 35–50 children whose ages ranged from 3 to 18 years. The small CHs housed six children whose ages ranged from 3 to 18 years. The average length of stay in institutions for children in all CHs in Japan is 4.9 years [1]; 2.7% of those children are orphans [2]. The environments of these large and small CHs are similar, and the CHs exist in the same area as the traditional families. Convenience sampling was used to select the CHs. A CH manager has parental authority over the children in their care, and thus were contacted as gatekeepers during recruitment. A general invitation letter was sent to gatekeepers that explained the scientific character of the survey, and the voluntary nature of participation in the study. Before the study began, all the CH managers gave written informed consent and gave their permission for their children to participate in this research.

Students from traditional families were recruited from an elementary school and a junior high school in Yokkaichi city, which is the main city in Mie prefecture (traditional family group). Both of these schools are located near the city center. Before the study began, the Yokkaichi City Board of Education and principals of the schools gave informed consent, and their permission for their students to participate in this research. All the students and their parents also gave written informed consent. Data were collected in July 2016. Students were recruited through their teachers while in class, using convenience sampling. Students were required to answer the questionnaire without any support, because in previous research, parents reported higher QOL for their children than their children did for themselves [17, 18].

### Measurement

School teachers gave the questionnaire booklets to the participants’ parents. CH staff gave the questionnaires to their resident participating students. All participants anonymously filled in the questionnaire by themselves. The questionnaire took approximately 10 min to complete, and was collected in closed envelopes by teachers or CH staff.

QOL was measured using the Kinder Lebensqualität Fragebogen KINDL®; [19, 20], which is translated from German as, Children’s quality of life questionnaire [21]. It was developed in Germany in 1994, and has been used worldwide to evaluate the health-related QOL of both ill and well children and adolescents in the general population. The total number of question items is only 24, which puts a reasonable demand on young participants [22]. The self-report kid-KINDL® Japanese Version for elementary and junior high school students also consists of 24 items equally distributed among the following six subscales: physical well-being, emotional well-being, self-esteem, family, friends, and school [13]. The validity and reliability has been well-established by previous research [13]. For children in a CH, the term “family” in the questionnaire referred to staff and other children in their CH. Each item addressed experiences over the past week and was rated on a 5-point Likert scale (1 = “Never”; 2 = “Seldom”; 3 = “Sometimes”; 4 = “Often”; 5 = “Always”). Mean scores were calculated for each of the six subscales for a total scale, and linearly transformed to a 0–100 scale [17].

### Statistical analysis

Complete case analyses were conducted. Welch’s *t*-test was conducted to compare differences in the mean QOL scores between children in institutional care and those in traditional families. This test was also used to compare mean QOL scores of large and small CHs. Significance level was set as *p* = .05. Cohen’s *d* was calculated as standardized effect size by dividing the mean differences between two groups with pooled standard deviations between those two groups. Statistical analyses were performed with R Commander version 1.6–3 and EZR 1.32 [23].

## Results

We obtained responses from 48 of 56 participants in the CH group (85.7% response rate) and from 168 of 243 in the traditional family group (69.1% response rate). Table 1 records characteristics of the students in each group: Mean age, age range, and gender.

**Table 1:** Participant demographics

	^a^Children’s Homes	Traditional families
	*n* = 47	*n* = 157
Age (years)		
Mean (SD)	10.7 (2.11)	11.6 (2.16)
Range	8–14	8–15
Sex		
Female	55.8%	54.1%
Male	44.2%	45.9%

^a^Children’s Homes were established by child social welfare law in Japan, and house orphaned, neglected, or abused children

**Fig. 1:**
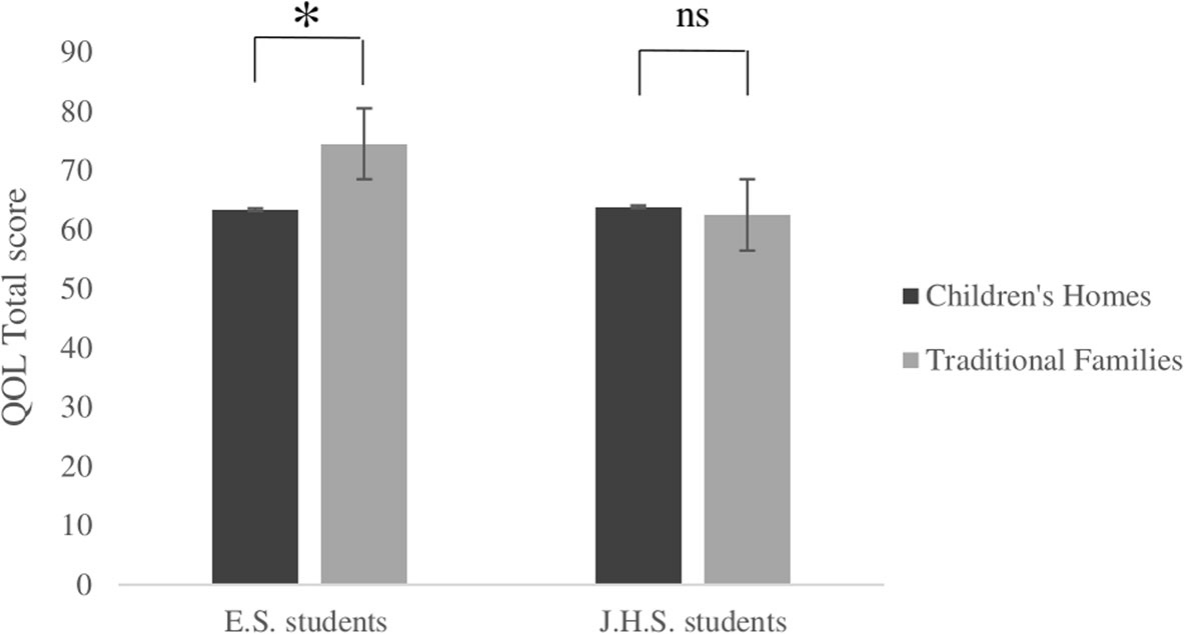
Quality of Life (QOL) total scores for elementary and junior high school students in Children's Home group and in traditional family group

As shown in Table 2, the mean QOL total score for the CH sample (63.54, *p* = .033) was statistically significantly lower than that for the traditional family sample (68.44). Mean scores for the emotional well-being (74.04, *p* = .049) and family (61.07, *p* = .001) subscales were also significantly lower in the CH group than in the traditional family group. Table 3 shows that mean total QOL and all subscale scores of the elementary school students in the CH sample (total QOL = 63.38, *p* = <.001; emotional well-being = 71.48, *p* = .004; self-esteem = 49.62, *p* = .005; family = 58.50, *p* = <.001; school = 54.23, *p* = .009) were statistically significantly lower than those of the traditional family sample (total QOL = 74.57; emotional well-being = 83.88; self-esteem = 64.17; family = 74.16; school = 66.76). Table 4 shows the QOL scores for the junior high school students in the CH sample (total QOL = 63.86, *p* = .73; physical well-being = 72.71, *p* = .19; emotional well-being = 79.00, *p* = .64; self-esteem = 36.75, *p* = .88; family = 66.07, *p* = .63; friends = 73.50, *p* = .76; school = 55.53, *p* = .26) and in the traditional family sample (total QOL = 62.54; physical well-being = 65.93; emotional well-being = 76.91; self-esteem = 37.81; family = 68.80; friends = 75.15; school = 49.45). There were no observed statistically significant differences between total QOL score and each of the six subscales. Figure 1 shows the mean QOL total scores for the elementary and junior high school students in the CH group and in the traditional family group. Table 5 shows the responses obtained from 15 children in small CHs (total QOL = 65.78, *p* = .40; physical well-being = 73.95, *p* = .58; emotional well-being = 74.26, *p* = .95; self-esteem = 46.70, *p* = .79; family = 63.01, *p* = .63; friends = 74.68, *p* = .58; school = 62.95, *p* = .09) and 32 children in large CHs (total QOL = 62.49; physical well-being = 77.17; emotional well-being = 73.93; self-esteem = 44.56; family = 60.17; friends = 70.92; school = 50.79). No statistically significant differences were observed between small and large CHs in either total QOL score or in each of the six subscales.

**Table 2:** Quality of life (QOL) scores and subscale scores for Children’s Homes residents and traditional family members

Variable	^a^Children’s homes *n* = 47		Traditional families *n* = 157		Effect size	*p*
	M	SD	M	SD		
Total score	63.54	13.26	68.44	14.88	0.23	0.033
Physical well-being	76.14	17.33	71.64	18.23	0.17	0.12
Emotional well-being	74.04	19.51	80.33	16.91	0.24	0.049
Self-esteem	45.24	25.98	50.74	25.21	0.15	0.20
Family	61.07	19.20	71.43	20.07	0.34	0.001
Friends	72.12	23.61	77.01	17.42	0.17	0.19
School	54.67	21.53	57.94	21.06	0.11	0.36

^a^Children’s Homes were established by child social welfare law in Japan, and house orphaned, neglected, or abused children. QOL was measured with the Kinder Lebensqualität Fragebogen (KINDL®) questionnaire, Japanese Version; SD = Standard Deviation; M = Mean

**Table 3:** Quality of Life (QOL) scores for elementary school students

Variable	Students from ^a^children’s Homes *n* = 31		Students from traditional families *n* = 77		Effect size	*p*
	M	SD	M	SD		
Total score	63.38	13.27	74.57	13.14	0.47	< 0.001
Physical well-being	77.91	16.70	77.58	17.55	0.01	0.92
Emotional well-being	71.48	20.77	83.88	17.06	0.40	0.004
Self-esteem	49.62	24.61	64.17	19.92	0.39	0.005
Family	58.50	18.55	74.16	17.83	0.48	< 0.001
Friends	71.41	25.57	78.94	17.83	0.23	0.14
School	54.23	22.78	66.76	19.17	0.36	0.009

^a^Children’s Homes were established by child social welfare law in Japan, and house orphaned, neglected, or abused children. QOL was measured with the Kinder Lebensqualität Fragebogen (KINDL®) questionnaire, Japanese Version; SD = Standard Deviation; M = Mean

**Table 4:** Quality of Life (QOL) scores for junior high school students

Variable	Students from ^a^children’s Homes *n* = 16		Students from traditional families *n* = 80		Effect size	*p*
	M	SD	M	SD		
Total score	63.86	13.67	62.54	14.12	0.08	0.73
Physical well-being	72.71	18.55	65.93	17.11	0.29	0.19
Emotional well-being	79.00	16.29	76.91	16.15	0.10	0.64
Self-esteem	36.75	27.23	37.81	22.99	0.03	0.88
Family	66.07	20.07	68.80	21.79	0.10	0.63
Friends	73.50	19.98	75.15	16.91	0.07	0.76
School	55.53	19.57	49.45	19.33	0.24	0.26

^a^Children’s Homes were established by child social welfare law in Japan, and house orphaned, neglected, or abused children. QOL was measured with the Kinder Lebensqualität Fragebogen (KINDL®) questionnaire, Japanese Version; SD = Standard Deviation; M = Mean

**Table 5:** Quality of Life (QOL) scores for children in small and large Children’s Homes

Variable	Children from small ^a^children’s homes *n* = 15		Children from large ^a^children’s homes *n* = 32		Effect size	*p*
	M	SD	M	SD		
Total score	65.78	11.58	62.49	14.03	0.15	0.40
Physical well-being	73.95	19.87	77.17	16.24	0.11	0.58
Emotional well-being	74.26	18.62	73.93	20.21	0.01	0.95
Self-esteem	46.70	24.69	44.56	26.92	0.05	0.79
Family	63.01	18.01	60.17	19.95	0.09	0.63
Friends	74.68	19.72	70.92	25.44	0.09	0.58
School	62.95	23.23	50.79	19.90	0.34	0.09

^a^Children’s Homes were established by child social welfare law in Japan, and house orphaned, neglected, or abused children. QOL was measured with the Kinder Lebensqualität Fragebogen (KINDL®) questionnaire, Japanese Version; SD = Standard Deviation; M = Mean

## Discussion

This study presents a comparison of the QOL of young people living in CHs with those in a traditional family home. Results showed that total QOL, emotional well-being, and family scores in the CH group were statistically significantly lower than those in the traditional family group. Previous research has shown that children aged five to 11 years old receiving child welfare services have significantly lower QOL than the general child population [24]. Moreover, that institutional care has a significant negative impact on QOL of children [11, 25, 26]. These trends correspond to our results. Critically, about 60% of children in CHs in Japan are abused [3]; their abuse typically begins before they enter an institution. This history of abuse, and associated experiences with mental health treatment (successful or not) may negatively impact these children’s QOL and thus help explain the differences in QOL scores in the present study.

While this study showed that the total QOL, emotional well-being, self-esteem, family, and school of elementary school children in the CH group were statistically significantly lower than those in the traditional family group, junior high school students in the CH group in the present study did not show any significant differences in their total scores for QOL and its subscales, compared with junior high school students in the traditional family group. These results are not consistent with previous studies of this generation or population, which found that the QOL of children and adolescents in institutional care was lower than those in a traditional family sample [27, 28]. In a previous study using KINDL®, QOL scores decreased from elementary to middle school students, especially in self-esteem [13]. A plausible reason for these declines when becoming a junior high school student is the accumulating stress of, for example, developmental tasks, pressure to improve school grades, interpersonal relationships, acquisition of basic beliefs and values for autonomy in adolescence [16]. Similarly, in the present study, junior high school students in the traditional family group rated their QOL lower than that of the elementary school students in the same group. However, as mentioned, junior high school students in the CH group did not rate their QOL lower than their peers in the traditional family group. Expanding on our explanation for the lack of difference in QOL scores between junior high school students across the two groups, we suggest that it is possible that students in the CH group may have been more anxious about their results being seen and associated with them by CH staff, despite the questionnaires being collected in closed envelops, and thus they may have self-reported higher scores. It is possible, too, that CH adolescents have been under more stress than their traditional family peers since their elementary school years, because of being removed from their homes. Therefore, the QOL scores of CH children do not change substantially from elementary to junior high school because their QOL is typically already low by the time they are in elementary school.

Many studies have reported on problems associated with children in institutional care (e.g., orphans) in developing countries such as Romania [11, 29–37]; however, Rutter et al. [8] reported that no research has compared the results from studies of Romanian orphans with other countries’ orphans. Thus, the foregoing studies have limited generalizability in terms of an understanding about which children are adopted in developed countries from deprived institutions in developing countries [38]. Rutter et al. [8] also reported that Romania’s institutions have few staff members, so the children in their care lack social interaction, which negatively affects the children’s mental and physical development. Previous investigations found that institutional conditions can improve child behavioral outcomes considerably by increasing caregiver interactions with children [39, 40]. Thus, caregivers-to-children ratios and caregivers’ interactions with children have important implications for children’s QOL. [39]. CHs in developing countries like Romania, moreover, face budgetary restrictions that limit the number of child-careers they can hire. In Japan, by contrast, CHs have much more financial freedom; the Child Welfare budget in Japan for 2014 was approximately $1 billion [41]. The Child Welfare Law in Japan states that for infants under 3 years old, a CH should generally provide one or more caregivers per two infants; for children over 3 years old, they should generally provide one or more caregivers per four children; and for those older than elementary school age, they should generally provide one or more caregivers per six children.. CHs in Japan hire clinical psychologists, social workers, nurses, nutritionists, cooks, and child care personnel to help raise and care for the children. Additionally, medical doctors are often employed part-time by some CHs [42]. Thus, children living in CHs in Japan can receive support from specialists (e.g., individual and group psychotherapy with clinical psychologists). Compared with many institutions in developing countries, the living environment in Japanese institutions is arguably better. Nevertheless, this study suggested that elementary pupils living in CHs have lower QOL than their peers who live in traditional families.

While no statistically significant difference was observed in the present study between small and large CHs in either total QOL score or each of the six subscales, caution should be used when generalizing to other settings and participants because of the small sample size. It is important to note that Child Welfare Laws in Japan allow CHs to hire many caregivers, irrespective of the size of the institution. This means that the care provided for children is of high quality even in large CHs; caregivers-to-children ratios are the same for small and large CHs, and thus would not explain any statistically significant differences between QOL of students in small versus large CHs. The UN has recommended that Japanese governments provide family-like settings such as are found in small CHs or foster families [6] for children who need social care. However, our results do not support this recommendation from the viewpoint of children’s QOL. Furthermore, unlike institutional care, foster care does not provide multiple professional staff members to care for children, but rather one or two parents; the staff in CHs, moreover, work rotating shifts, while foster caregivers are unlikely to provide such constant time commitment. Actually, parental authority can be very strong in Japan [43]; parents ultimately have the authority, and may even abuse their own children. Therefore, it is often difficult for abused children to adapt to foster parents. However, the QOL of children living in foster care in Japan was not within the scope of this study; further research should carefully investigate this aspect of child care in Japan.

This study contributes to the literature in that it is the first to investigate the QOL of children living in CHs in Japan, using the KINDL® as a measurement instrument. The results were further strengthened by having the children themselves respond to questions about their QOL, rather than relying on the proxy reports of child caregivers. However, several limitations of this study should be discussed. First, the sample size was relatively small, which limits the generalizability of the findings, and the convenience method of sampling potentially affects the representativeness of the participants. Second, this research did not adjust for potentially confounding factors such as, for example, maltreatment, life history, and intelligence quotient (IQ), because the Research Ethical Committee in the Mie University School of Medicine did not approve using such records. Third, the traditional family sample may have been skewed because parents of dysfunctional families are less likely to agree to answer questions posed in the KINDL®. This could help explain the higher QOL found in the traditional family sample.

## Conclusions

The findings presented support previous research showing that the QOL of elementary school students living in CHs is significantly lower than that of their peers in traditional families. However, this difference was not observed among junior high school students. This contrast suggests that QOL changes with age. Future research is needed to evaluate the determinants of QOL among all generations and family contexts.
